# Official Report upon Intrathoracic Surgery

**Published:** 1919-01

**Authors:** 


### AN OFFICIAL REPORT

**Report upon Intrathoracic Surgery.**

From Laboratory of Surgical Research, Central Medical Départment Laboratory, American Expeditionary Forces.

To : Brig. Gen. J. M. T. Finney, M. C., U. S. A., Chief Consultant in Surgery, American E.F.

In accordance with your direction, an effort has been made to standardize procedure for the surgical treatment of thoracic injuries: first, by determining in the Laboratory the anatomical and physiological reactions fundamentally significant in repair; and, second, through the practical application of the principles thus established to the treatment of the wounded. The experimental work was done in the Laboratory of Surgical Research, and the clinical service was rendered in various hospitals of the forward areas reserved for the care of the non-transportable wounded.

It is impossible to submit a final report, because laboratory observations are incomplete and late clinical results are unavailable. An unexpectedly early disassociation of those engaged in this investigation has also made it impracticable to prepare illustrations, and to make proper acknowledgment of benefits derived from the work and writings of others.

*Problem.*

Therapeutic methods were desirable to reduce the immediate and remote mortality, to extend effective surgical treatment to some of the more severely wounded, hitherto considered hopeless, and to secure, in a larger proportion an earlier and more complete functional recovery of those convalescing from thoracic injuries. The requirements were a safe and simple method of performing thoracotomy; an operative technic for pulmonary repair that would provide immediate reinflation and re-establishment of some degree of respiratory function, and, later, assure maximum pulmonary inflation and elasticity.

This problem consisted of the following factors :

(1) Parietal repair — primary healing must be assured.

(2) Limitation of pleurisy — reduction in intensity, extent and duration.

a) Elimination of pleural irritation.

b) Determinate factors in pleural resistance.

c) Lung repair, preserving its elasticity.

d) Reinflation of lung — re-establishment of intrathoracic negative pressure.

e) Immobilization of affected side of chest.

f) Drainage with conservation of normal negative pressure.

(3) Anaesthesia — administration of nitrous oxide and oxygen so as to control intrapulmonary pressure.

*Experiments* :

An epidemic of rabies, which occurred during the experimental stage, made it imperative that all stray dogs be destroyed. The mayor of the city in which the Laboratory was located gave his permission to use the animals that remained unclaimed at the pound for experimental purposes. The dogs were invariably operated upon in ether or nitrous oxide narcosis, and were protected against unavoidable distress by a free use of morphine before and after the operation. The facilities provided for this work by the Central Medical Department Laboratory and the Research Department of the Red Cross were so excellent that they would have been welcome in front area hospitals.

*Parietal Repair* :

Failure to obtain air-tight parietal healing after thoracotomy leads to inevitable distress, frequently to death. Immediate and lasting union of the parietal pleura is more important than that of any other structure. It is the surest protection against complete breakdown through extension of inflammation from without inward, or, more important still, from within outward. Opportunity for satisfactory repair is assured if the pleura is united surface to surface, as in intestinal suture, and if an adequate blood supply is provided by the avoidance of tension upon sutures.

Rib resection permits thoracotomy with the least damage from the standpoint of healing. After incising the anterior periosteum in the mid-line of the rib, it is carefully reflected so as to avoid injury to intercostal vessels or nerve. When the rib is resected a similarly placed incision in the posterior periosteum opens the chest. This loose periosteum, the inner reflection lined with pleura, immediately retracts and forms at the margins of the incision a welt of thickened tissue admirably adapted for holding sutures without interfering with essential blood supply. Contraction of the intercostal muscles, with respiration, produces immediate separation of wound edges after thoracotomy. These margins cannot thereafter be maintained in approximation without undue suture tension; consequently the ribs just above and below the incision must be brought abnormally close together. This rib approximation can be accomplished by passing a suture about them, and tying it with sufficient tension. Absorbable sutures, various types of catgut and kangaroo tendon, proved to be incapable of resisting this strain until there was firm healing. Silk, linen and cotton are inferior to wire. Heavy silver wire, or that made from aluminum bronze alloy, proved to be satisfactory.

It is essential that mattress sutures be used in the pleural closure to guarantee surface approximation of the serosa. No rib denuded of its periosteum should be permitted to appear within the pleural cavity, as such potential foreign bodies lead to pleurisy or recurrent hemothorax. This is avoided by stripping the periosteum a short distance beyond the line where the rib is divided, and so placing the marginal stitches that the pleural pucker begins beneath and lateral to the rib ends.

Layer by layer the muscles and fascia are closed with interrupted stitches, so that they are accurately but not too snugly approximated. Because of the abnormal rib approximation there is some dead space to fill, but also redundant tissue with which to fill it. The principle of imbrication variously applied serves this purpose admirably. At intervals these interrupted stitches should include the layer next below. This reinforces, prevents dead space, and, most important of all, limits the extension of infection. A separate closure of the superficial fascia is profitable.

Dogs are peculiarly susceptible to empyema. In a series of thoracotomies this method of closure broke down only in consequence of extension from infected pleurisy, and when tension on the parietal pleura stitches, resulting from inadequate rib fixation, caused the parietal pleura suture line to give way. Similar observations have been made repeatedly at human necropsies, and have indicated the necessity of guarding against this danger.

a) *Limitation in Degree and Extent of Pleurisy.*

All foreign bodies are irritants, and all irritants in the presence of recent infection are undesirable. Blood itself is sufficiently irritant to the pleura to provoke a sero-fibrinous reaction. This reaction is sufficiently intense in the presence of hemothorax to unite the visceral and parietal pleura by fibrinous adhesions in the abnormal relationship consequent upon pulmonary compression and dislocation. Full blood is more irritating than defibrinated blood, which in turn is more irritating than blood serum. Fibrin could not be introduced intrapleurally without thoracotomy, which invalidated direct comparison. Almost certainly it is more irritating than full blood, hence the desirability of removing all the blood and coagula. Full blood coagulates in the chest in rapidity and in completeness directly proportionate to the degree of tissue injury, which determines the conversion of prothrombin into thrombin. Hemothorax induced with a minimum tissue injury coagulates very slowly, because blood platelets and leukocytes degenerate slowly under conditions so slightly abnormal, which is a partial explanation for the belief that blood does not clot in the pleural cavity. Removal of other foreign bodies, bone, metal, bits of liver, etc., is even more essential. Flushing the pleura with solutions, saline, Dakin's solutions, etc., gives an appearance of greater macroscopic cleanliness, but reduces resistance to an extent that makes irrigation unjustifiable. Moreover, the slightest additional irritation to the pleura increases subsequent effusion.

b) *Determinate Factors in Pleural Resistance.*

Area for area the healing reactions of the pleural and peritoneal serosa are histologically and physiologically similar, if not identical. The peritoneum is accepted as one of the best healing and most resistant tissues in the body; the pleura is known to have much less resistance. An anatomical and physiological comparison is illuminating.

The parietal pleura and peritoneum differ little in extent and in richness of blood and nerve supply. The visceral peritoneum is much greater in extent than the visceral pleura (the entire peritoneum is estimated at 98 o/o of the skin surface), a material portion of which is upon the omentum. Resistance to infection seems to be proportionate to the extent of visceral serosa and the richness of its blood supply. It is impossible for dead space to occur intraperitoneally, and immobilization of intraperitoneal viscera can be made virtually complete with the administration of opium. The more complete the immobilization of the peritoneum in the presence of intraperitoneal irritation, the less the consequent effusion and diffusion, and the more rapid (4 to 6 hours) is the formation of fibrinous adhesions. These adhesions, inevitable in the healing of serous membranes, are desirable to protect local resistance.

The peritoneum has been shown to have the capability of exuding serum at the rate of 5 o/o to 6 o/o of the body weight an hour, and of absorbing in almost the same rapidity. Under certain conditions intraperitoneal injections are virtually equivalent to intravascular. In the pleural cavity the potentiality for rapid effusion seems to be proportionately as great as the peritoneal, but the powers of absorption are much less. An amount of normal saline that would be taken up in a few minutes by the peritoneum may be shown with the aid of the fluoroscope to require 48 to 72 hours to disappear from the pleural cavity.

The three conditions precedent to smooth healing, elimination of irritation, physiological rest, and increased blood supply, are equally effective in promoting intrapleural resistance. It is because of the inter-dependence of circulatory and respiratory functions upon pleural integrity that its preservation assumes such great significance. Elimination of irritants needs no further discussion. Physiological rest cannot be made as nearly perfect intrapleurally as intraperitoneally, without affecting the even more important factor of circulation. Lung can be immobilized in two ways, by deflation or compression, and by inhibiting costo-diaphragmatic movement. Lung deflation is produced with some degree of pneumothorax, which means dead space and decreased resistance. Lung compressions can be attained only by the presence of a foreign substance in the pleural cavity, and this, too, means decreased resistance. Both of these conditions cause pleural irritation and pulmonary deflation. Pleural irritation, in addition to causing increased effusion and pleural thickening, provokes inflammation in the adjacent lung parenchyma. This cortical pneumonia varies in degree and in extent with the duration and intensity of the pleurisy. It is prone to organization rather than to resolution, and the resulting increase in peripheral scar tissue is in part the explanation of the difficulty in securing subsequent pulmonary inflation. It is also a possible explanation of the decrease of failure of pleural effusions to be absorbed. Protracted deflation causes a retraction of elastic tissue, vessels and nerves, similar to the neurovascular-fascial contraction, consequent upon the fixation of joints in flexion. The blood supply to lung and pleura is proportionate to extent of physiological inflation.

Experimental evidence indicated that pneumothorax so rapidly absorbed from normal pleura was slow to disappear in the presence of an acute pleurisy. The effects of the introduction of various fluids intrapleurally were studied to determine the feasibility of a clinical application of this principle of immobilization of injured lung during the early period of healing. Normal saline was found to be the least irritating, and to be absorbed with desirable rapidity in 48 to 72 hours. However, where salt solution was put into the pleural cavity of thoracotomized animals, the parietal pleural suture line broke down in each instance. This was attributable to the force of respiratory and cardiac impulses transmitted through the fluid, and to the direct effect of the saline solution upon an adequate deposition of fibrin. Moreover, both of these methods produced increase in the rate and the distress of respiration. Inhibition of costo-diaphragmatic movements could with safety be induced in two ways. The rate and depth of respiration could be decreased with full doses of opium, which is effective when given in subtoxic amounts, by decreasing metabolism and consequently reducing oxygen requirements, with little or no depression of the respiratory centres under this condition. Or the diaphragmatic excursion could also be eliminated by blocking the phrenic nerve. It was found that injections of magnesium sulphate and of quinine and urea hydrochlorate were ineffective. Cocain was superior to novocain, in that less accurate infiltration of smaller amounts was effective. The resulting paralysis lasted from 4 to 5 days, and was succeeded by a gradual return of diaphragmatic function. The effect upon the diaphragm, determined fluoroscopically, was an immediate inhibition of contraction on the injected side. The only motion, and that very limited, was due to contractions from the contralateral side, transmitted through the central tendon. The relaxation of the paralysis permitted intra-abdominal pressure to raise the level of the diaphragm on the affected side, but insufficiently to cause serious pulmonary deflation. Diaphragmatic paralysis carried with it a decrease in costal excursion, so that the total reduction of intrapleural motion was considerable. These results were the same whether the phrenic was injected in the neck or in the chest, and no untoward results such as bronchitis or pneumonia were observed. This is precisely the mechanism that nature has developed in combating pleural pneumonia. When these principles were applied to thoracotomized animals, the beneficial effects were definite. A small group of dogs were not given morphine after operation. Their convalescence was less certain, longer, and the distress increased. A series in which the phrenic was injected showed a remarkable freedom from distress and a reduction in the amount of post-operative pleural effusion. Still more significant was their general condition on the 4th or 5th day, when the diaphragm resumed its function. This stage of convalescence corresponded to the 10th to 14th day in those animals subjected to identical operations, but in which no phrenic injection had been made.

In order that the three cardinal conditions upon which healing depends may be established after thoracotomy, the following precautions must be observed : reduction in irritation by the least mechanical trauma; protection of the serosa against exposure and drying; preservation of lung elasticity; accurate pleural approximation; the establishment and maintenance of normal negative intrapleural pressure; and the restriction of respiratory function for the first few days; c) *Lung Resection and Surgical Repair.*

Resection or deep incision of lung parenchyma must qe made so that the resultant impairment of air and blood circulation will not interfere with possibilities of healing and of the return of function. Studies were made of the anatomy involved by injecting the bronchial and pulmonary arteries with a Barium suspension, and then making radiograms of excised, inflated lungs. Branches of the bronchi and pulmonary arteries are given off at nearly right angles to the parent stems near the hilus, but as the vessels extend toward the pleura, the angles of departure of the branches become progressively more acute. Physiological effects were determined by arterial and bronchial ligature in the living animal. Ligation of the bronchial artery causes atrophy of the part supplied, which on section shows a peculiar slaty palor. Ligation of the pulmonary artery was followed by necrosis, which affected the periphery (pleura) primarily, and extended thence backward to the hilus, with a characteristic convoluted regularity of outline. Ligation of a bronchus to any lobe produced a purulent bronchitis, and later broncho-pneumonia with pleurisy. This was attributed to infection of secretions retained under pressure, due to interference with normal drainage. Ligation of very small bronchial branches is productive of no serious consequence. A simple practical method of determining when an obstructed bronchus endangers the corresponding parenchyma is provided by the use of positive intrabronchial pressure. So long as the parenchyma can be inflated it is safe. An obvious danger of infection from the air and mucus forced through the severed bronchi was found experimentally to be negligible. A suspension of B. Prodigiosus was insufflated intratracheally, positive pressure established, and subsequent culture taken from the bronchi after incising the lung — these results were negative. Lycopodium granules similarly insufflated could not be demonstrated upon incised lung surface.

The lung tissue up to the margins of surfaces to be united after operation must be viable and capable of function. Parenchymatous surfaces must be adequately approximated to insure healing without interference with function. Lung parenchyma is the equal of any tissue in the capacity for repair and for spontaneous hemostasis. All branches of the bronchial artery, distinguishable by spurting red blood under higher pressure, must be ligated. Also the larger pulmonary vessels should be tied. Severed bronchial twigs are disclosed by the bubbles escaping from them, because of the positive pressure anaesthesia described later. Those that are visible should be ligated.

*Approximation* of the lung surfaces must be achieved without producing avoidable induration from either the repair or from subsequent cicatrization, so that there may be little effect upon the elasticity of the organ. This closure must permit immediate lung function; the pleural surfaces must be in apposition for air-tight closure. These conditions prohibit the use of mass sutures. Fine stitches, as many as are required to give satisfactory approximation, are to be placed with the lung in full inflation. They should be placed superficially and tied just tightly enough to produce approximation. Surprisingly few are required to accomplish this closure. Pleural surfaces are to be united by a continuous mattress stitch of the Cushing type : this can be effected with little puckering, and with an air-tight suture line. When complete, one knot is the only foreign body exposed upon the surface.

d) *Inflation of Lung to Re-establish Normal Negative Pressure.*

Inflation of the lung during operation has essential advantages. It facilitates surgical manipulation by bringing the operative field nearer the surface, thereby reducing need for traction. Traction transmitted to the mediastinum is prone to produce an immediate and considerable drop in the systematic blood pressure. Inflation is the only way by which hemorrhage and escape of air can be controlled under conditions approaching the normal.

It is possible to inflate and deflate the lungs when the chest is opened without affecting the systematic blood pressure, because of the powers of compensatory adjustment in the rate of flow, amount, and distribution of blood in the pulmonary circulation. Inflation up to normal limits does not apparently throw an increased burden upon the right heart. Air-tight parietal closure, while the lung is thus distended, permits an immediate re-establishment of approximately normal, negative, intrapleural pressure, since the residual pneumothorax is so limited as to be negligible.

e) *Immobilization of Affected Side of Chest.*

Injury alone suffices to restrict costal excursion upon the side involved. If the injury be severe enough to provoke pleurisy, hemorrhagic or serous, there may be also a limitation in the diaphragmatic excursion. This reflex reduction in motion can be supplemented but little by the use of pressure bandage. A snug bandage has good effect upon the wound healing. If immobilization of greater degree is desirable it can be attained by posture and by nerve block.

f) *Drainage.*

Experiments on the treatment of infected pleural injuries simulating war wounds had just been started when orders came for duty in the forward area. The principles, but not the means of drainage, had been determined. An acceptable drain must be inserted in the costo-phrenic sinus so as to offer no obstruction to full pulmonary inflation, and where the consequent pleural reaction would be least harmful. It must permit air-tight insertion, and have a simple automatic one-way valve. This method would protect the degree of inflation and negative pressure obtained at operation, and tend to increase both if they were subnormal. Thus it would be applicable both in primary and secondary drainage, and when inserted with positive pressure anaesthesia make feasible free drainage of early empyema before adhesions were present. Ultimately a satisfactory means was devised. An " L " shaped piece of glass tubing, 12-15 mm. in diameter with its arms 3 to 4 cm. long, was completely covered by thick rubber tubing. The rubber tubing was cut flush at one end, and here the flat valve from a box respirator was slipped over and securely tied in place. The rubber tubing extended beyond the other end far enough to reach the level of the crest of the diaphragm and prevent occlusion.

A short, low rib resection permitted this tube to be inserted with the end from which the rubber tubing projected on the inside, and turned upwards. Air-tight layer closure that lasted for 4 or more days was easily accomplished. This simple apparatus fulfilled requirements already given, and under conditions that reduced individual attention to post-operative patients almost to the vanishing point.

g) *Anaesthesia.*

A simple method of giving nitrous oxide and oxygen, utilizing tank pressure, to secure needed degree of inflation, was devised by Captain Gwathmey. A full pre-operative dose of morphine made possible the induction of deep analgesia, without increasing the nitrous oxide and oxygen rates above 3 to 1, which Lieut. Col. Cannon's experiments had proved to be the limit of safety in the presence of shock. Lieut. Cattell's observations had indicated that morphine thus given had value as a prophylactic agent against oncoming shock, and therapeutic value when given early in the presence of shock. No untoward result from depression of the respiratory centre was noted.

Animal experiments showed clearly that administering anaesthesia under tension, particularly when the chest was opened, was dangerous if the gas or ether was given in increased concentration. It also demonstrated that thoracotomy with all incidental manipulations, such as dislocation and operation upon lungs, should be performed under the primary stage of anaesthesia. Manometric observations showed that when the pressure present in the mixing bag reached 8-16 mm. Hg. it sufficed to distend the lungs completely; that degree of pressure is present when the bag fluctuated little during inspiration. Since this degree of tension in the bag produced an intrapulmonary pressure that was well within the limits of safety for dogs, the manometer was not deemed a necessary adjunct for human use.

A safe sequence in practice was found to be as follows: After the effect of the pre-operative hypodermic of morphine was present, administrations of pure oxygen under no tension were started. Then very gradually the pressure was increased, and the administration of nitrous oxide started. Rapid induction of the anaesthesia was undesirable. Avoidance of excitation and the producing of gradually increasing inflation were essential. During the operation the proportions of the gas-oxygen mixture and the pressure transmitted to the trachea were varied to meet conditions. After the parietal pleura was closed the amount of nitrous oxide was gradually reduced; last of all, oxygen under pressure was continued until the patient was conscious.

The American Red Cross nitrous oxide apparatus, perfected by Capt. Gwathmey and adopted by the Army, fulfilled every requirement. This apparatus provides a mask that can be rendered relatively air-tight by close approximation to the face, an escape valve, a mixing bag close to the inhaler, and a rough gauge for estimating the proportion of the gasses.

Intrapulmonary pressure was raised by increasing the rapidity of the flow of gasses from the tanks, and by increasing the pressure upon the face piece. It was lowered by decreasing the rate of flow of the gasses or by releasing the valve or decreasing the pressure which held the face piece in place. Thus, any degree of desirable inflation or deflation was promptly available to meet operative requirements. In general the degree of pressure utilized was that best suited to the animal or man under operation.

This method gives all practical requirements for intra-thoracic surgery without necessitating deep anaesthesia for the introduction of intra-tracheal or endopharyngeal tubes. Moreover, its safety and ease of control has removed the chief obstacle to a wider application of surgical therapy.

h) *Practical Application.*

So closely related is the physiology of man and dog, in spite of the gross anatomical differences in the pleura and mediastinum, that the principles established experimentally were directly applicable to the wounded. The human chest and lungs are far easier to manipulate, and more resistant to infection. Methods had to be varied to meet conditions, but, so far as was determined, there was no change in principles indicated from the end results.

Granted proper facilities, satisfactory triage, early transportation and a trained personnel, 100 % of chest wounds demanded surgical treatment *but not necessarily thoracotomy.* This radical statement is made from the standpoint of the wounded individual, and takes no account of battle conditions and the requirements of evacuation. It is based upon the following facts : Experience has demonstrated the impossibility of eliminating bone injury without exploration in through and through bullet wounds of the chest, for example. Under the analgesia described it is easy to follow a bullet tract down to the entrance or exit from the thoracic cavity. Not infrequently an unsuspected rib fracture, even complete and comminuted, is disclosed. Further investigation, even when an unbroken but perforated rib has presented, will commonly show that the inner compact portion of the rib has been splintered, loose fragments driven into the lung or attached fragments turned into the pleural cavity. Such injuries may be accompanied by an inconsiderable amount of hemothorax, or it may develop that an injured intercostal artery is accountable for the bleeding. Since the pressure in the systemic circulation is 6 to 8 times higher than it is in the pulmonary circuit, and the spontaneous control of bleeding in the thorax has been developed to combat the lesser pressure, an accurate control of such hemorrhage is of utmost importance. Surgical treatment of these lesser injuries is simple; some rib resection is indicated. The positive pressure will have returned the wound in the lung close to the parietal wound, through which it can be examined. If the lung injury be considerable, enough rib must be resected to give sufficient room to repair the lung damage. If it be a dry puncture, it may be disregarded as the positive pressure would have started anew bleeding from any dangerous source. If the lung hole be bleeding, it can be readily closed with a purse string suture of the visceral pleura, after gently withdrawing a cone of lung through the parietal opening. Should the injury to the parieties and to the lung be slight, and the hemothorax considerable, the uncoagulated portion of the latter can be removed by introduction of the aspiration nozzle (to be described later). The orifice of entrance or exit which gives the best approach should be used. After the lung is allowed to collapse a fairly satisfactory baling out is made possible. If the patient be in a dangerous condition, the irritation from the clots which remain may be compensated for by the saving of time and manipulation, attendant upon a more radical operation.

The wound of exit should also be explored, because of the danger of rib damage. Here, too, for no evident reason, bone fragments can be driven backward into the lung.

Three means of parietal closure can be employed. If a considerable opening has been made, a routine closure, with rib stay of wire, is indicated. If the parietal opening be less extensive, visceral pleura may be stitched into the defect, or the same may be plugged with a transplanted muscle surface. In any case, the closure should be air-tight, and the last sutures tied, after full inflation is attained. Experience has shown that injuries thus treated repair well, and that parietal debridement of even bullet wounds is not contra-indicated. A large hemothorax and hemopneumothorax of unusual proportions demand radical treatment though caused by through and through bullets without bone involvement. A large hemothorax provokes serious pleural reaction and pulmonary compression. Immediate aspiration is contra-indicated, because fresh bleeding may result, and because part of the blood and all of the clots will fail of removal. Immediate intervention is indicated for reasons previously given. Large pneumothorax indicates the possibility that a large bronchial branch has been opened, and the danger of empyema from such a source.

The preceding groups may comprise 40 %, or even more, of the chest injuries, depending upon the type of conflict. If to them be added groups of similar injuries produced by small shell fragments, they will together comprise those deemed by many as best treated expectantly. This conservative opinion is the result of the dangers attendant upon thoracotomy performed with open methods of anaesthesia, or without the use of nitrous oxide as above described. There is a group of very grave chest injuries that during the stress of battle must be denied surgical treatment. During periods of less pressure these patients should be given every chance of recovery offered by operation, even though there is reason to question the probability of immediate survival.

Again, the analgesia from nitrous oxide and oxygen is the big factor, so little is the distress it causes. If surgical intervention offers the faintest chance of recovery, improbable by expectant measures, the individuals deserve the chance. The fetish of statistics, and particularly the immediate mortality rate, has had too great an influence upon the attitude of clinicians functioning as medical officers. The recovery of no individual should be jeopardized by want of surgery, but, on the contrary, every service should be rendered by giving even the worst risks any chance operation may offer.

There is another group made up of those having sucking wounds, large retained foreign bodies, liver injuries and obvious compound fracture of ribs or scapula that indisputably are suitable for surgical treatment. The methods of operating upon individuals showing such injuries may be divided into two types.

When the parietal wound is so severe that its treatment is going to contra-indicate any further intervention, or when the intrathoracic injuries can best be approached through that pathway, adequate rib resection should be made at such a site. This method of approach has distinct advantages. It obviates additional incisions, leads directly to a part at least of the intrathoracic damage, reduces the duration of operation, and eventually reduces the total adhesive pleurisy by bringing the parietal wound into contact only with the visceral. It has the disadvantage, if the wound be high, low or near the median line anteriorly or posteriorly, of restricting the field of intrathoracic exploration. Further, the tissues about the incision are bruised and infected, and cannot be expected to heal ideally. Neither can the deep closure of such a wound be entirely satisfactory. Unfortunately, this method is demanded in a considerable proportion of the severely wounded.

A more satisfactory method is to excise and repair the projectile wounds according to the methods indicated in the discussion of through and through bullet injuries. The chest is then opened by an incision placed ideally; or the thoracotomy may be performed first, and the results of the intrathoracic examination would determine if more need be done.

A section of the 4th rib, long enough to provide space for an easy insertion of a closed hand, should be removed from its postero-lateral aspect. This resection can be effected without cutting across muscle fibres that will be needed directly in closing the deeper wound. The latissimus dorsi can be drawn backward, and the pectoralis major drawn forward, after dividing its inferior lateral fibres close to the attachment, to secure adequate relaxation. The serratus magnus can be split between its fibre bundles. The opening thus obtained will allow inspection or palpation of the entire cavity, unless there are extensive adhesions. No mechanical rib spreader is needed, and should not be used, as such instruments crush the intercostal muscles, and interfere with the blood supply to the pleura. Additional exposure can be obtained only by rotating the third and fifth ribs. This rotation is best accomplished by lifting directly upward with broad-bladed abdominal retractors.

With this exposure it is possible to operate on virtually any part of non-adherent lung. After partial deflation a lobe can be grasped with a Tuffier forceps, guided towards the opening in the chest wall, and made to present therein without traction simply by increasing the degree of inflation. If a diaphragmatic injury is suspected, resection of the 5th rib will make its repair possible. After such an operation an accurate closure is easy, and the healing satisfactory.

Experience in taking care of the wounded taught other principles worthy of record. Skin incisions should not be made completely at first, unless there is little doubt about the deeper injuries. When these have been determined through an incomplete incision, the balance can be adapted to circumstances. As a rule, simple straight incisions are the best. At times flaps must be reflected, as the skin and fascia may be the only covering that can be provided for the pleural closure, which in itself may be incomplete.

In general, muscle splitting methods comparable to the gridiron incision for appendectomy, are most satisfactory. In this way debridement of successive layers can be done, cut muscle bundles kept distant from the site of injury, and smooth muscle surfaces preserved for final approximation. It is well to remember that recovery cannot take place without parietal healing, and parietal healing can occur after considerable bruising and soiling, even if a very extensive debridement is omitted. This applies even to the erector spinae group of muscle.

Bone injuries are handled with greater difficulty. Ribs may be resected at will, and should be sacrificed always to give proper approach and at times to permit of better closure. Spinal injuries, if the cord is intact, should be treated as any other compound fracture; converted, if possible, into a simple fracture by primary suture. If there is a cord lesion present only sucking wounds should be operated, and these only to secure parietal healing. Scapula may be tilted, or resected, leaving as much periosteum and margins for muscular attachments as conditions permit. The parietal pleura must be protected. A hole in it is about as easily repaired as a hole in a drum head, and there is about the same facility in its mobilization. Defects that cannot be repaired without tension should be filled either by stitching visceral pleura to the margin, or, if low enough, by suturing to the diaphragm. If this is not possible, a smooth muscle surface should be applied.

When access to the thoracic cavity has been obtained, it is wisest to remove free blood at once, to prevent any further clotting. Any form of suction apparatus suffices. There should be a glass or a metal tube long enough to reach any part of the chest, and having its distal end covered with a coarse wire bulb to prevent pleura and large clots from plugging the intake. Just before closure, all the clots and excess fibrinous exudate should be removed with gauze, if this is impossible manually.

Foreign bodies must be removed unless embedded in ribs, so that extraction requires another procedure unwarranted by the patient's condition. It is particularly essential to extract projectiles from the mediastinum, or when they are in close contact with large vessels.

Lung wounds may be closed safely with less resection than similar injuries to other tissues, because of its exceptional powers of resistance and repair. More than a slight trimming of shaggy margins should be reserved for these instances in which the blood and air circulation are irrevocably impaired. This presents the most difficult question to decide in the chest problems. When does hemorrhagic infiltration cease to be a recoverable lesion and amount to a hemorrhagic infarction? Some lungs in which this infarction had been suspected to have occurred were resected, and recovery followed. In other patients partial lung resection, followed by death, failed to show at autopsy that the resection of itself had contributed to the fatality. In no instance where resection was absolutely indicated by the anatomical condition, and prevented by the general condition, did a survival occur. These fatalities were associated with a particularly virulent empyema. Only a little less baffling was the question of collapse. A part of the lobe, usually the lower, would be found atelectatic. The associated injury was generally severe and involved overlying ribs. Such a collapse could not be inflated during operation. None such was resected, because of depressed general condition. All that were recognized died. At autopsy the same portion was still collapsed, the balance of the lung slightly emphysematous, and an empyema was present. Apparently the pleura overlying the segment of such abnormal lung ceases to function defensively if it has not become an actual foreign body.

Adhesions should be severed or torn only when necessary to make urgent repair. The tissue thus exposed is little resistant. All of these perforated chests are infected. The control of purulent pleurisy depends entirely upon the defense of the tissues exposed upon pleural surfaces. Chronic pulmonary emphysema sufficient to prevent normal deflation so reduces pulmonary circulation as to impede healing of lung parenchyma, and should be removed as the most dangerous factor in producing subsequent inflammation. Bullet and shell fragments larger than a few mm. in diameter can usually be palpated, and easily extracted.

The larger the bronchial branch that is opened, the greater the danger of infection, particularly when there is ready access from the mucosa to the pleura. If such an opening cannot be made air-tight, it should be sealed with a pleural graft.

Injuries to the diaphragm are readily repaired. Liver injuries introduce bile into the pleura, where it is more harmfully irritant than in the peritoneum. The same sutures that reunite a torn diaphragm can be made to unite, or at least to fix the liver laceration. Transpleural drainage works beautifully where it is not needed. Injuries to other abdominal organs may at times be repaired by enlarging the left-sided diaphragmatic defect.

Primary drainage is indicated in the presence of an established pleurisy, particularly where it is associated with a liver injury and consequent bile irritation, or when resulting from urine which has seeped into the chest from renal laceration, or when complicated by a sucking wound of many hours duration. It is also probably safer to drain at operation, when wide resection or extensive collapse make impossible an elimination of pneumothorax, and when there is reason to doubt the viability of lung or pleura. It must be remembered that whereas drainage of the general peritoneal cavity is impossible, drainage of the pleural cavity is feasible and safe, if properly done. The old dictum " when in doubt, drain ", as applied to the peritoneum, came to be " when in doubt, don't drain ". It is quite possible that the reverse may prove to be the outcome in the pleural cavity.

Indications for secondary drainage will be considered in the discussion of post-operative treatment.

i) *Pre-operative Treatment.* An individual with a chest wound is particularly in need of physical and psychic rest. This can be provided by an early administration of a full physiological dose of morphine (1/3 to 1/2 grain). Other benefits to be derived from this drug have been enumerated.

Care of the wound includes accurate hemostasis, particularly of the intercostals. Suckers should not, as a rule, be provisionally sutured. Such a suture may lead to a considerable emphysema, with consequent reduced resistance in the tissue about the wound, may give a false sense of security, which leads to delay in operation, and assures some degree of pulmonary deflation. Unless there is a brisk uncontrollable hemorrhage from inside the chest, it is better to apply a large pad to the chest wall, completely covering the wound, fix it in place with adhesive, and then place a firm bandage, or a swathe, about the thorax. Besides decreasing distress, such a dressing acts as a suction valve permitting the egress but not the ingress of air, and, by encouraging inflation, protects the pulmonary circulation.

Uncomplicated chest wounds are notably unlikely to foster shock. On the other hand, they are frequently productive of a high venous pressure, resulting from disturbed pulmonary circulation. Therefore, when shock is present any intravenous injections must be given with special care not to overburden an already embarrassed right heart. Particularly important is a determination of the venous pressure under these conditions. If possible, transfusion should be done after pulmonary inflation has been established with the positive pressure of the anaesthetic. Even when there is no shock, if there be a large hemothorax, a blood transfusion is indicated when hemostasis is accomplished. A grave anaemia may be masked by an unusual distribution of blood, because of compression of the pulmonary circulation.

j) *Diagnosis and Classification.* Physical and fluoroscopic examination furnish fairly accurate data from which to determine urgency of operation. Projection of the wounds of entrance and of exit, or of the wound of entrance and the location of a retained missile (unless it was free in the pleural cavity) gave the course of the projectile approximately, and identified the structure probably involved and to some extent indicated the degree of damage. Especial care was taken in attempted determination of the presence and nature of bone injury. Urgency of operation as an immediate life-saving procedure is primarily to be considered, but ultimate functional recovery is also a factor in the following classifications :

Group 1. *Hemorrhage.* This group includes those suffering from the acute anaemia of recent hemorrhage, and those who are still bleeding. Measures to be adopted must include immediate hemostasis, the safe-guarding against secondary hemorrhage, blood transfusion, and, when possible, repair according to established principles.

Group 2. *Sucking wounds.* If conditions permit, this type should be treated like any other chest injury, plus closure of the parietal defect. Otherwise, simple closure, or a closure plus primary drainage, is demanded for immediate safety, relegating other measures for the future to determine.

Group 3. *Bone involvement.* Every penetrating chest wound should be explored down to the pleural wound. Bone fragments are the most dangerous foreign bodies to both pleural and pulmonary healing, immediate and remote.

Group 4. *Pneumothorax.* This uncomplicated is rare, and due to injury of a large bronchus. Closure of this defect is demanded to prevent lethal complications.

Group 5. *Hemothorax of symptoms.* Those of this group suffering from acute anaemia have been considered in Group 1. The balance show respiratory and circulatory embarrassment due to pressure. As a rule, they have better chance of recovery if given immediate surgical care, permitting accurate hemostasis and repair of visceral injuries.

Group 6. *Foreign bodies.* The presence of a foreign body is sufficient to indicate operation for its immediate removal when it is large (and then there will usually be additional indications), when it is in close proximity to vital structures, or when lying free in the pleural cavity. All foreign bodies should be removed incidentally to operations for other reasons, when this is compatible with the immediate safety, and if the removal requires no manipulations that will impair functional recovery.

Group 7. *Hemothorax of signs.* Simple hemothorax without symptoms. If limited in amount it should be treated expectantly, until there develops signs of infection or a rapid increase in amount, when radical treatment is indicated. If the hemothorax is large, and particularly when dense clots are demonstrable fluoroscopically, radical treatment promises earlier and more complete recovery with slight, if any, increased danger. A decision as to the treatment to be given to moderate sized hemothorax must depend on many factors, preference being given to conservatism.

Group 8. *Through and through chest wounds*, from bullets without any of the previous indications, or similar wounds from small shell fragments, are usually non-operative types, under ordinary conditions, and always under battle pressure.

k) *Post-Operative Care.*

From a functional standpoint the first 48 hours following thoracotomy under positive pressure anaesthesia contrasted most favorably with those performed under open ether. Dyspnoea and distress were vastly reduced, and post-operative shock cut to a minimum. Indeed, not infrequently the patient left the table in better condition than before operation. The degree of functional return effected by the elimination of post-operative pneumothorax contributed largely to this result. Further limitation of post-operative effusion, reduction in the incidence of pneumonia, and elimination of contralateral pulmonary collapse were remarked. These cases were observed for 4 to 12 days, but, unfortunately, under existing circumstances, the end results could not be determined.

Morphine, by reducing the respiratory rate, and by affording rest, is a prime requisite in the post-operative treatment of chest wounds. The dosage must be guided by the condition and reaction of the individual. The indications of its efficacy are freedom from cough, pain and distress.

Posture is an over-rated point in post-operative treatment. Any position producing a lateral curvature of the spine, with the concavity toward the affected side, will lessen the extent of respiratory movement on this side. The simplest means to produce this end, pillows, etc., are sufficient. The degree of elevation of a patient depends entirely on his condition and comfort.

Post-operative effusion inevitably consequent upon thoracotomy may be met by one of two divergent plans of treatment. The dry method attempts to keep the pleural surfaces in continuous contact by repeated aspiration. This facilitates the formation of adhesions and hastens a return of lung function. Such a plan is impracticable in field work, so that the expectant plan was followed. Pressure signs, or signs of infection of the effusion, were the primary indications for aspiration. An argument for this plan lies in the rest afforded by splinting the lung and restricting diaphragmatic excursion in the early period. Pleural effusions do not assume recognizable proportions until the second day, so that for this period pleural resistance is not materially affected by the presence of the fluid The secondary indication for aspiration is the failure of this fluid to be absorbed.

Early return of the maximum degree of pulmonary function is an important feature in post-operative treatment. Neither active nor passive lung exercise should be attempted until the patient is afebrile. If, when begun thereafter, they are followed by recrudescence in temperature, such exertions should be discontinued until the patient is again afebrile. Measures involving some degree of personal attention or interest, such as blowing exercises, or the simple expedient of cutting down the respiratory rate to a minimum (by a watch) promises the greatest degree of success. Inconclusive observations on the use of positive pressure attained by giving oxygen through an anesthetic inhaler in this stage of the post-operative care were made. Exercises bringing into play the muscles of the thoracic cage should be introduced as soon as the patient is able to get out of bed.

1) *Complications.*

Post-operative pneumonia is commonly contralateral and of the lobular or bronchial type. Infrequency of its occurrence in this series obviously has some relation to the use of positive pressure and of gas and oxygen in anaesthesia. It is possible that the inordinate respiratory effort induced by post-operative pneumothorax of the open method of anesthesia predisposes to, or actually excites an insufflation pneumonia. The treatment of this pneumonia is largely supportive. Cold air is contraindicated, unless the patient's condition be particularly robust.

Contralateral collapse did *not* occur in this series. Positive pressure was probably effective in eliminating this complication.

Post-operative empyema bears a definite relationship to the nature and condition of the wound, and its duration before operation. The condition of the pleura at the time of operation is of great pronostic value. A shaggy, fibrinous exudate, or a dirty pleura argue against an uncomplicated convalescence. The incidence of empyema, in patients operated upon later than 24 hours after the receipt of the injury, mounts very rapidly. A sudden increase in the fluid level, the patient's condition, together with signs of sepsis, and the bacteriologic and microscopic study of fluid removed by exploratory puncture, all determine the final diagnosis of empyema. If the organism be staphylococcus or pneumococcus, and if the patient's condition be good, repeated aspirations are permissible and often suffice. In the presence of streptococcus empyema, or that due to members of the anaerobic group of bacteria, immediate drainage is desirable.

Post-operative empyemas are productive of limiting adhesions in inverse ratio to the virulence of the causative bacteria. Consequently, the urgency for drainage is overbalanced by the contraindication of pulmonary deflation, unless some positive pressure anesthesia be employed and a suction type of drainage introduced. Using such methods, immediate secondary drainage can be established with a diagnosis. It must be remembered that, under field conditions, close individual attention is impossible and repeated aspiration, as in civil practice, cannot be performed.

m) *Wound Healing.*

When fairly good surgical technic is possible, primary sutures of chest wounds give better results than wounds of a similar type elsewhere, because of the possibility of complete immobilization.

During a period when there was pestilence of flies, an undue number of such wounds suppurated. These infections tended to remain superficial, and delayed only that part of wound healing. The percentage of empyema was also highest at this time.

In a series of 120 operations involving the chest wall, in not a single instance did gas gangrene develop, and no virulent streptococcus infection occurred.

n) *Results.*

Statistics showing mortality rates in war injuries can never be convincing, because of the multiplicity of factors affecting the condition of the wounded. The series here analyzed is made up from some men wounded when in good condition, in warm dry weather, and given early treatment, but of more wounded of the opposite category. The work was done in field and mobile hospital units; the surgical facilities varied from wretched to excellent. Battle conditions permitting, no selection of cases was made to eliminate the bad risks. Any chance of operative benefit was given, and very gratifying recoveries resulted when fatalities were to be expected. This plan must carry with it a high mortality rate (29.6 o/o). Five deaths occurred upon the operating table, and twelve within 18 hours after operation. Thus, 63 o/o of the total mortality (27) occurred in patients too weak to withstand the strain of operation. Among these there were very few who might have recovered if treated expectantly, but there were more who did recover, who would have died if not given surgical treatment.

The operations were performed by Lieut.-Col. J. L. Yates and Major W. F. Verdi. The same methods were employed, and virtually identical results were obtained.

The results of 91 penetrating wounds of the chest treated by thoracotomy are analyzed in Tables I and II.

TABLE I

PENETRATING WOUNDS OF THE CHEST TREATED BY THORACOTOMY

Total cases . . . . . . . . . . 91 Total deaths . . . 27 (29.6 o/o)

*Nature of missile.*

| | | Deaths caused. |
|---|---|---|
| Bullet . . . . . . . | 39 (42.8 o/o) | 11 (40.8 o/o of total deaths. 28.2 o/o of group wounded by bullets). |
| Shell . . . . . . . | 52 (57.1 o/o) | 16 (59.2 o/o of total deaths. 30.7 o/o of group wounded by shell). |

*Group I.* — Chest alone involved :

| | | Deaths. |
|---|---|---|
| Uncomplicated . . . . . . . . . . . . . | 57 | 6 (10.5 o/o) |
| Profound shock. . . . . . . . . . . . . | 7 | 5 (71.4 o/o) |
| | 64 | 11 (17.1 o/o) |

*Group II.* — Chest involved, together with :

| | | Deaths. | |
|---|---|---|---|
| Abdomen . . . . . . . . . . . . . . . . | 9 | 6 | (66.6 o/o) |
| Other serious wounds . . . . . . . . . . | 14 | 7 | (50 o/o) |
| Added profound shock (to multiple wounds). | 4 | 3 | (75 o/o) |
| Totals. . . | 27 | 16 | (59.2 o/o) |

No further discussion of this Table will be attempted, except to call attention to the interesting fact that though chest wounds were more commonly caused by shell fragments, the percentage of deaths was proportionate to those caused by bullets.

In Table II a grouping of this series of 91 thoracotomies is attempted on the basis of the indications of operative urgency. The sub-grouping into " simple " and " complicated " has reference to the entrance of multiple factors, such as sucking wound plus bone involvement, for example, into the decision. Overlapping, therefore, occurs in the complicated sub-headings.

TABLE II

A GROUPING OF THE SAME ACCORDING TO A PLAN OF OPERATIVE URGENCY

| | No. | Deaths. | o/o Mortality. |
|---|---|---|---|
| I. *Hemorrhage acute anemia* . . . . . . . | 1 | 1 | 100 |
| II. *Sucking wounds* : . . . . . . . . . . . | | | |
| Simple. . . . . . . . . . . . . . . . . | 3 | 0 | |
| Complicated . . . . . . . . . . . . . . | 26 | 10[1] | 38.4 |
| III. *Bone involvement* : | | | |
| Simple. . . . . . . . . . . . . . . . | 27 | 6 | 22.2 |
| Complicated . . . . . . . . . . . . . . | 42 | 14[2] | 33.3 |
| IV. *Pneumothorax* — from large bronchus injury . . . . . . . . . . . . . . . . | 0 | 0 | |
| V. *Hemothorax of symptoms* : | | | |
| Simple. . . . . . . . . . . . . . . . . | 4 | 0 | |
| Complicated . . . . . . . . . . . . . . | 13 | 6 | 46.1 |
| VI. *Foreign Body* : | | | |
| Simple. . . . . . . . . . . . . . . . . | 6 | 0 | |
| Complicated . . . . . . . . . . . . . . | 4 | 0 | |
| VII. *Hemothorax of signs* : | | | |
| Simple. . . . . . . . . . . . . . . . . | 2 | 0 | |
| Complicated . . . . . . . . . . . . . . | 1 | 0 | |
| VIII. *Through and through without signs* : | | | |
| Simple. . . . . . . . . . . . . . . . . | 0 | 0 | |

1. Includes one spinal wound, one abdominal wound and one gas gangrene.
2. Includes 5 abdominal wounds, one femur and one section of vena azygos major.

Particular attention is drawn to the fact that no thoracotomy was performed on a simple, uncomplicated through and through wound of the chest. Individuals suffering from such wounds, therefore, are not included in the 91 cases. Further, it will be remarked that this series includes only those penetrating wounds of the chest treated by thoracotomy, and none of the group of non-penetrating chest wounds operated without thoracotomy.

In conclusion we must express our grateful appreciation of help and encouragement received. To you, Sir, we are indebted for the opportunity to study this problem, for practical suggestions and sound advice. We wish to thank the officers and enlisted men connected with the Central Medical Department Laboratory for their co-operation, particularly Col. J. F. Siler, Director of Laboratory Service, A. E. F., whose willingness and capacity to be helpful is unlimited. The American Red Cross, through the Research Department under Col. Alexander Lambert, has given essential assistance by furnishing facilities and equipment, both in the Laboratory and in field hospitals. We are greatly indebted to Major J. M. Steiner for his help and suggestion, and to Captain M. A. Blankenhorn for the benefits of his unusual experience derived from service in the B. E. F. under Col. A. B. Soltau.

Miss Anne Barnard, A. N. C. and Miss Anna Fitzgerald, A. N. C. have unfailingly shown the ability, energy and devotion to duty typical of the highest ideals of the American trained nurse. Sgt. 1st cl. P. W. Vestal, who has served with us from the first, and Pvt. C. Baronian, detailed more recently, have at all times been competent and willing.

